# Effect of Fine Size-Fractionated Sunflower Husk Biochar on Water Retention Properties of Arable Sandy Soil

**DOI:** 10.3390/ma14061335

**Published:** 2021-03-10

**Authors:** Łukasz Gluba, Anna Rafalska-Przysucha, Kamil Szewczak, Mateusz Łukowski, Radosław Szlązak, Justína Vitková, Rafał Kobyłecki, Zbigniew Bis, Michał Wichliński, Robert Zarzycki, Andrzej Kacprzak, Bogusław Usowicz

**Affiliations:** 1 Institute of Agrophysics, Polish Academy of Sciences 4, 20-290 Lublin, Poland; a.rafalska@ipan.lublin.pl (A.R.-P.); k.szewczak@ipan.lublin.pl (K.S.); m.lukowski@ipan.lublin.pl (M.Ł.); radoslaw.szlazak@gmail.com (R.S.); b.usowicz@ipan.lublin.pl (B.U.); 2 Institute of Hydrology of the Slovak Academy of Sciences, 841 04 Bratislava, Slovakia; vitkova@uh.savba.sk; 3 Department of Advanced Energy Technologies, Faculty of Infrastructure and Environment, Częstochowa University of Technology, 42-201 Częstochowa, Poland; rafal.kobylecki@pcz.pl (R.K.); zbigniew.bis@pcz.pl (Z.B.); michal.wichlinski@pcz.pl (M.W.); robert.zarzycki@pcz.pl (R.Z.); andrzej.kacprzak@pcz.pl (A.K.)

**Keywords:** available water content (AWC), biochar, sandy soil, soil water retention, sunflower husk

## Abstract

Biochar application has been reported to improve the physical, chemical, and hydrological properties of soil. However, the information about the size fraction composition of the applied biochar as a factor that may have an impact on the properties of soil-biochar mixtures is often underappreciated. Our research shows how sunflower husk biochar (pyrolyzed at 650 °C) can modify the water retention characteristics of arable sandy soil depending on the biochar dose (up to 9.52 wt.%) and particle size (<50 µm, 50–100 µm, 100–250 µm). For comparison, we used soil samples mixed with biochar passed through 2 mm sieve and an unamended reference. The addition of sieved biochar to the soil caused a 30% increase in the available water content (AWC) in comparing to the soil without biochar. However, the most notable improvement (doubling the reference AWC value from 0.078 m^3^ m^−3^ to 0.157 m^3^ m^−3^) was observed at the lowest doses of biochar (0.95 and 2.24 wt.%) and for the finest size fractions (below 100 µm). The water retention effects on sandy soil are explained as the interplay between the dose, the size of biochar particles, and the porous properties of biochar fractions.

## 1. Introduction

Biochar is a porous material produced by the thermal carbonization of biomass in the limited presence or absence of oxygen. It can be made of a variety of feedstock materials based on agro-forestry biomass and industry organic residuals, including wood, crops, husk, sludge, and manure. Different feedstock and process conditions can yield biochars with a diversity of physicochemical properties. Typical studies on biochar cover its application as a fuel, a remedy for polluted soils, and a soil-amendment material for CO_2_ sequestration improving soil quality for better growth of crops. The latter area of study is important from the perspective of agriculture, especially in the light of climate change. A canonical example of biochar-enriched soil is Terra Preta in the Amazon Basin. It contains an average amount of organic carbon corresponding to 50 Mg ha^−1^ of biochar and is well known for its excellent fertility [1,2]. In contrast, the typical nearby soils contain an order of magnitude less carbon and are less fertile. It took many years for the residents to develop the Terra Preta soil in the Amazon Basin. Nowadays, the achievement of a similar effect in a shorter time is desirable; however, this is complicated considering the variety of different soils and biochar types. It depends on many factors, e.g., numerous physicochemical properties and characteristics of both biochar and soil.

In certain conditions, biochar can improve the physical properties of soil for better crop yield by modifying the texture, structure, porosity, pore size distribution, and density [1,3,4]. Furthermore, biochar amendment can support biochemical properties by improving the availability of nutrients (K^+^, P^+^, and Ca^+^), increasing the amount of total organic carbon (TOC), decreasing soil acidity [1,5,6,7], and stimulating biological activity in soil [8]. Due to its porous structure, biochar can modify the fundamental hydro-physical parameters of soil, e.g., pore-size distribution and water flow [9], influencing the water retention curve of soil [10], and, consequently, the available water content (AWC) for crops. These features can be also dependent on the dose and particle size distribution of biochar, considering the type of feedstock and pyrolysis conditions. Thermally treated biomass can acquire diversified particle size distribution compared to the source material. The size of biochar particles can be further changed by post-pyrolysis mechanical treatment (e.g., sieving or grinding). Different size fractions of biochar obtained in the same process can have a varied composition and properties [11,12]. Various biochar size fractions in soil can influence, for example, hydraulic conductivity [10], water retention [4,13], soil aggregate stability [4], permanent willing point, field capacity [14,15], and microbial retention [16].

The specific reason for biochar application in agriculture is the prevention of drought. The problem of agricultural drought and related water shortages in soils and the demand to increase crop production have rendered studies on improvement of soil properties essential. In particular, the soil water retention property plays a significant role in soil management from an agricultural perspective and defines AWC for plants. Lately, increasing attention has been paid to the improvement of water retention in sandy soils in order to enhance fertility in areas with poor soil quality. Commonly, sand-textured soils have relatively high hydraulic conductivity and low retention of water and nutrients, which in drought conditions inhibits efficient growth of plants compared to soils with finer textures [17]. Although biochar does not markedly improve the properties of soils containing a moderate percentage of sand, it can be a remedy for the vulnerabilities of sandy soils by improving their physicochemical properties to support plant growth [18,19,20]. Considering hydraulic properties, typical biochar amendment to sandy soil can lead to an increase in AWC [21,22]. However, the effectiveness of the amendment may also depend on the dose and application of a certain particle size fraction [4,13,18,23]. The typical biochar grain size is below 2 mm [21,22]. There are few studies in which biochar contains only such fine fractions as <0.5 mm [4,13,14,23,24], and only one using size fraction below 63 µm [25].

Although notable improvements in AWC after biochar amendment were reported in the case of loamy sand soils (or loamy sands), there are only a few reports on a significant increase in AWC in sandy soils. De Jesus Duarte et al. [13] obtained a maximum AWC increase by 0.061 m^3^ m^−3^ of soil moisture after introduction of 0.46 wt.% of biochar with a grain size between 0.15–2 mm into tropical forest sandy soil. Biochar size fractions below 0.15 mm and above 2 mm were less efficient in increasing AWC of their samples. A similar size fraction as in the study conducted by De Jesus Duarte et al. [13] was used by Quin et al. [26]: the fraction of 0.25–2 mm and 1 wt.% added to sandy soil resulted in an increase in the AWC value by 0.01 m^3^ m^−3^ of soil moisture compared to the value for the unamended reference. In turn, Głąb et al. [23] showed a maximum increase in the AWC value by 0.061 m^3^ m^−3^ of soil moisture after addition of 4 wt.% of 0–0.5 mm biochar to sandy loam soil. A fine fraction of biochar (0.3–0.47 mm) was also applied by Suliman et al. [24], giving a 0.079 m^3^ m^−3^ increase in the soil moisture content. Similarly, the meta-analysis on biochar effects presented by Edeh et al. [27] suggests application of the ϕ < 2 mm fraction of biochar for AWC improvement. Therefore, the tailoring of the particle size distribution of biochar before amendment can lead to a maximization of expected benefits. Furthermore, new routes should be explored to enhance biochar-related effects in cases where the application of unfractionated or roughly sieved pyrolyzed biomass does not bring an expected positive outcome. Nevertheless, there are still only few detailed studies on how certain fractions of biochar modify the biological or physicochemical properties of soils [28].

The literature shows that the influence of biochar amendments on hydrophysical properties in soils may be hard to predict due to many factors that are often difficult to identify. Thus, there is a need for better understanding of biochar effects in soil to improve the productivity and sustainability of agri-food production. In this paper, we show studies on sunflower husk biochar as a potential soil amendment that is still poorly investigated. We attempt to improve hydro-physical properties of arable sandy soil by applying different doses of biochar and we focus on the influence of its size fraction, which is not common in the literature. In particular we use particles of biochar with ϕ < 50 µm, 50–100 µm, and 100–250 µm. The amendment of soil with such small biochar particles often leads to a reduction in the amount of plant-available water in the soil due to strong bonding of water molecules in small pores [13]. On the other hand, the role of small biochar particles depends on its properties and like in Ref. [25], may increase the AWC value. Here, we verify that approach using fine size fractions of sunflower husk biochar.

## 2. Materials and Methods

### 2.1. Soil

The study was conducted using Podzol soil sampled from a research test-site containing an arable field located in Sęków (51°21′ N 23°16′ E) [29]. Soil samples were taken from the top-soil layer (0–25 cm); then, they were air-dried, homogenized, and sieved (<2 mm). The textural soil parameters (sand, silt, and clay) were determined using the Casagrande aerometer (Soil Science Society of Poland, Warsaw, Poland) method with Prószyński modification, based on studying changes of the density of soil suspension during the sedimentation process at constant temperature [30]. As a part of this procedure, the organic matter content (OM) was obtained by hydrogen peroxide digestion. The OM content was also determined with the Tiurin digestion and titration method [31]. The pH values were obtained with a pH meter using H_2_O and KCl solutions using 1:10 soil suspension.

### 2.2. Biochar

Biochar was produced from sunflower husk pellets in a pilot lab-scale reactor constructed at the Częstochowa University of Technology. The core part of the reactor consisted of a 3 meter-long retort (steel tube) with a 0.1 m diameter (internal), which was electrically heated (4 sections) from the outside. The temperature of the thermal treatment of the feedstock was controlled and maintained at the desired level along the retort. The feedstock was transported along the retort via a steel screw conveyor which allowed for easy and simple adjustment and control of the feedstock residence time and its transport velocity within the retort. The control of these parameters was crucial for the production of biochar with the desired parameters (carbon content and porosity) [32]. The pyrolysis process of the sunflower husk was carried out at inert atmosphere with no external gas at 650 °C for roughly 15 min. The particle size analysis was carried out by sieving 150 g of the biomass sample for 30 min in an automatic Retsch AS 200 sieve analyzer (Haan, Germany).

The contents of *C*, *H*, *N*, *S*, and *O* in the samples were determined with a Leco TruSpec CHNS (St. Joseph, MI, USA) elemental analyzer according to ISO 29541:2010 and ISO 19579:2006 standards. The contents of *C*, *H*, and *N* were carried out at 950 °C, while the *S* content was investigated at 1350 °C to assure the decomposition of all sulfur compounds in the sample. The oxygen content in the biochar was calculated as follows (Equation (1)):

(1) $$O\left(\%\right)=\;100-\left(C_{d}+H_{d}+N_{d}+S_{d}+Ash_{d}\right)$$

where the superscript *d* denotes the sample in a dry state and the *Ash*_d_ is the ash content in the sample determined form weighing of the residue after 4 h thermal treatment at 575 °C in an electrically-heated furnace (SNOL-1.6.2.5.1/13, Narkunai, Lithuania).

The pore size distribution, porosity, and pore volume were investigated with a mercury porosimeter (Quantachrome PoreMaster 33 (Quantachrome Instruments, Boynton Beach, FL, USA), which allowed the automatic investigation and analysis of the sample morphology and structure. The apparatus is operated with nitrogen as the gas being compressed. The apparatus enables an investigation of the the distribution of both meso- and macropores and is operated at low pressure (1.5–350 kPa; suitable for the pore range of 950–4.26 µm) and at high pressure (0.140–231 MPa) in order to determine the pore sizes between 10.66 µm and 0.0064 µm (6.4 nm). The pore diameters were estimated using the Washburn Equation (2):

(2) $$D=\frac{-4\gamma_{Hg}\cos\theta }{P}$$

where *γ_Hg_* is the surface tension of mercury, *θ* is the contact angle between liquid mercury and the pore wall, and *P* is the applied pressure.

The imaging of the sunflower husk biochar sample topography was performed with JEOL JSM-6610LV (Jeol Ltd., Tokyo, Japan) scanning electron microscope (SEM) using 20 kV of accelerating voltage for the electron beam.

The powder X-ray diffractometry (XRD) studies were performed with the Seifert 3003 TeT (GE, Boston, MA, USA) diffractometer equipped with a cobalt lamp (voltage 30 kV, λ_Co_ = 1.7902 Å, filament current 40 mA).The detector scintillation counter SZ 20/SE was applied to investigate the phase composition of the biochar sample.

### 2.3. Soil-Biochar Samples

Like the soil samples, the biochar was first sieved through a <2 mm stainless mesh before amendment. Next, the sieved biochar was fractionated (<50 µm, 50–100 µm, and 100–250 µm). The sieving procedures were performed using meshes and a vibratory sieve shaker (Fritsch Analysette 3, Idar-Oberstein, Germany). The soil-biochar mixtures used in the study consisted of two series of samples. One group with sieved biochar with a grain size below 2000 µm included 0.95, 2.24, and 4.76 wt.%, which corresponded to 20, 50, and 100 Mg ha^−1^, respectively. The other series consisted of soil samples amended with three biochar size fractions—below 50 µm, 50–100 µm, and 100–250 µm at the same doses as before. Additionally, the soil samples were mixed with a high amount of 9.52 wt.% of fractionated biochar, which corresponded to 200 Mg ha^−1^. An unamended reference sandy soil sample was investigated as well. The soil-biochar mixtures and the control (soil without biochar) were prepared in triplicate. Air-dried samples were weighed to obtain their bulk density values.

### 2.4. Water Retention Curves and Analysis

The water retention curves (WRC) of the soil-biochar samples were measured using the pressure plate method equipment (Soilmoisture Equipment Corp., Santa Barbara, CA, USA). Initially, the soil samples placed in metal cylinders (volume 100 cm^3^) were saturated with distilled water until a thin film of water was seen on the surface. Then they were drained at a small negative-pressure head *h*_w_ = −2 cm in a sand tank, which corresponded to a negligibly higher value than pF = 0. The samples were weighed (saturated weigh and “−2 cm” weigh) and transferred onto a semipermeable ceramic plate placed in a pressure chamber. To preserve good contact between the soil samples and the porous plate, the pressure-plate was slightly wetted. The container was sealed to be airtight, and the measured pressure was applied. When no water outflow from the apparatus was observed, it was assumed that the sample reached the equilibrium. Air from the pressure container was released; the samples were weighed, carefully returned to the pressure-plate, and the pressure was applied. The process was repeated six times at different pressures: 0.06, 0.15, 1.0, 1.58, 4.8, and 15.0 bar, which corresponded to pF = 1.85, 2.2, 3.0, 3.2, 3.7, and 4.2, respectively. After the last weighing, the samples were oven-dried and finally weighed. Then, the soil water contents were evaluated with the gravimetric method [33]. Every sample was analyzed in three repetitions, and the WRC data are averaged values. However, some samples in triplets revealed extraordinary results; hence, they were not taken into account in averaging.

Based on the experimental data, the WRC values were fitted using the unimodal van Genuchten model (VG) [34]. The dependence between soil moisture (θ) and matric potential (h) reads as (Equation (3)):

(3) $$\theta =\;\theta_{r}+\;\left(\theta_{s}-\theta_{r}\right){\left[1+{\left(\alpha \left|h\right|\right)}^{n}\right]}^{-m}$$

where $\theta_{r}$ and $\theta_{S}$ are the residual and saturated volumetric water contents, respectively. The parameter m = 1 – 1/n, where n is dependent on the pore-size distribution. The matric potential of air entry is described by α. The quality of VG model fits is measured by the coefficient of determination *(*$r^{2}$). The AWC values were calculated as a difference between the values of volumetric water contents at pF = 2.0 (field capacity) and pF = 4.2 (permanent wilting point) determined using van Genuchten function (Equation (3)) and the fitted parameters.

## 3. Results

### 3.1. Soil and Biochar Parameters

The analyses of the unamended soil texture revealed 90% of sand (2 mm–0.05 mm), 9% of silt (0.05 mm–0.002 mm), and 1% of the clay fraction (<0.002 mm), confirming the sand textural class. Before the biochar addition, the soil contained 1.77% of organic matter. Parameters of soil used for those studies are shown in Table 1.

**Figure 1:**
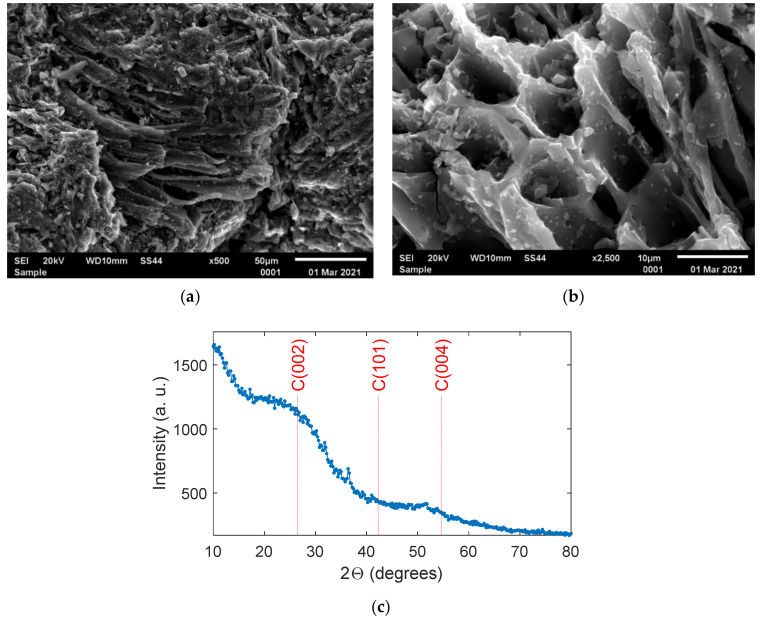
Scanning electron microscope (SEM) images of the sunflower husk biochar sample for: (**a**) ×500 and (**b**) ×2500 magnification. (**c**) Powder X-ray diffraction pattern of the biochar sample with marked the angular positions of graphite crystallographic planes reflections.

The topographies of the sunflower biochar samples obtained from SEM are shown in Figure 1a,b for magnifications 500× and 2500×, respectively. The images revealed a typical morphology of pyrolized biomass that confirm the porous structure of the sunflower husk biochar. The elemental analysis showed that the sunflower husk biochar consisted of 85%, 3%, 1%, <0.1%, and 2% of C, H, N, S and O, respectively. The level of ash content was 8.1%. The results of XRD of biochar, shown in Figure 1c, unveiled the broad features indicating completely amorphized structure. The main feature is the peak marked as C(002) in Figure 1c at 2Θ = 20°–30° that originates from randomly oriented aromatic carbon sheets in amorphous carbon structure [35]. The feature at 2Θ = 45°–55° may correspond to overlapped C(101) and C(004) plane reflections that are possibly broadened and shifted due to a high density of defects.

**Figure 2:**
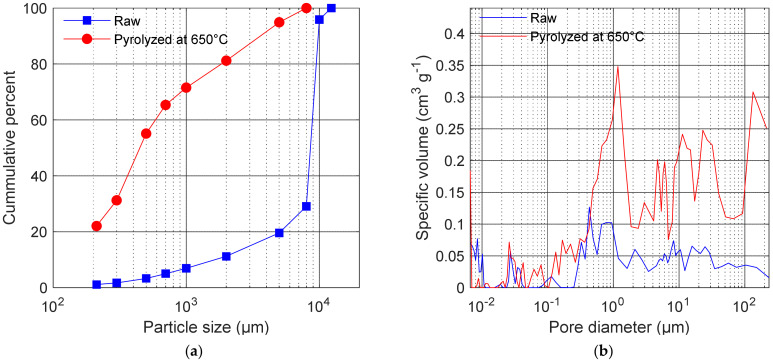
(**a**) Cumulative particle size distribution of the raw sunflower husk pellet (squares) and thermally processed material at 650 °C (circles). The fine fraction of biochar (ϕ < 212 µm) accounts for approximately 20% of the total volume. (**b**) Results of the pore diameter vs. specific pore volume for the raw and pyrolyzed (at 650 °C) material. After the thermal processing, the biomass increased its specific pore volume, which was the most evident above the 0.3 µm pore diameter.

The particle size distribution of the feedstock material (sunflower husk pellet) and biochar after the pyrolysis is shown in Figure 2. The size distribution of raw pellet was dominated by approximately 10 mm particles. After thermal processing, the finer fractions considerably increased their contribution to the cumulative distribution. Additionally, before the amendment of the soil samples, we measured the composition of the finer fractions (below 250 µm) used in this study (also shown in Figure 2).

The raw sunflower husk pellet had 8.8% and 0.115 cm^3^ g^−1^ of total porosity and total specific pore volume, respectively. Due to pyrolysis at 650 °C, the biochar total porosity and total specific pore volume increased to 15.5% and 0.652 cm^3^ g^−1^, respectively. Detailed results on the specific volume of pores in the raw and processed material are shown in Figure 2b. There was a significant increase in the volume values across the nearly entire range of the measured pore diameters due to the pyrolysis process. The largest increase was observed for the pore diameters above 0.4 µm.

**Figure 3:**
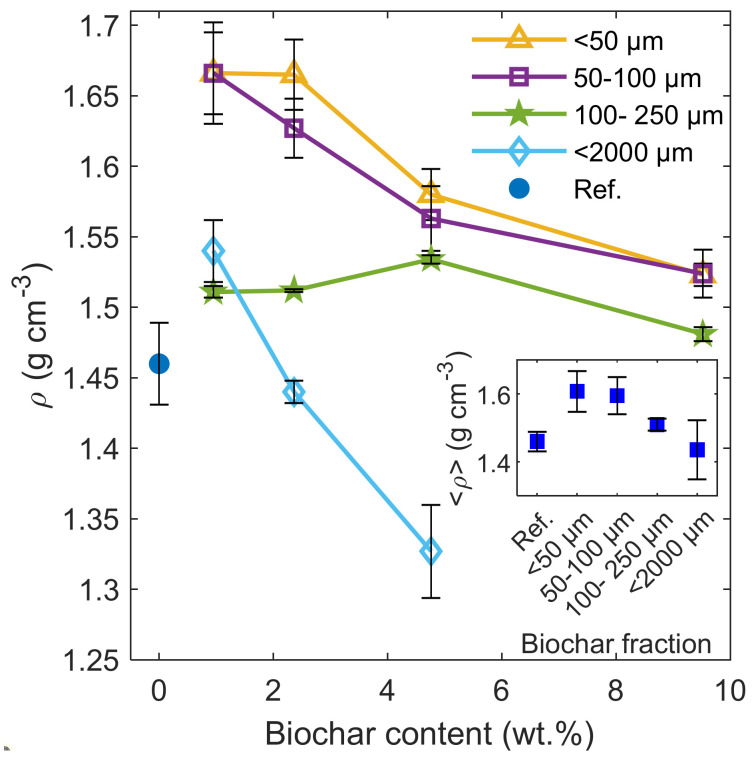
Bulk density (ρ) of the soil-biochar samples together with the standard deviation, compared to the reference value measured for the bare soil sample. The inset picture shows the dose-averaged bulk density ($<\rho >$ ) for the studied biochar fractions.

The biochar amendment caused an increase in the bulk density (ρ) of the soil-biochar samples treated with the lowest dose, compared to the reference value (Figure 3).

The most significant increase was recorded for the finest fraction of biochar (ϕ < 50 µm), which is presented in the inset plot of Figure 3 showing dose-averaged bulk density ($<\rho >$) values across all the biochar fractions. In the case of fractions with ϕ < 250 µm and ϕ < 2000 µm, after an initial increase, the ρ value was found to decrease at the higher biochar contents. Only the 100–250 µm fraction exhibited the maximal ρ value at the 4.76 wt.% biochar content.

### 3.2. Water Retention Properties

**Figure 4:**
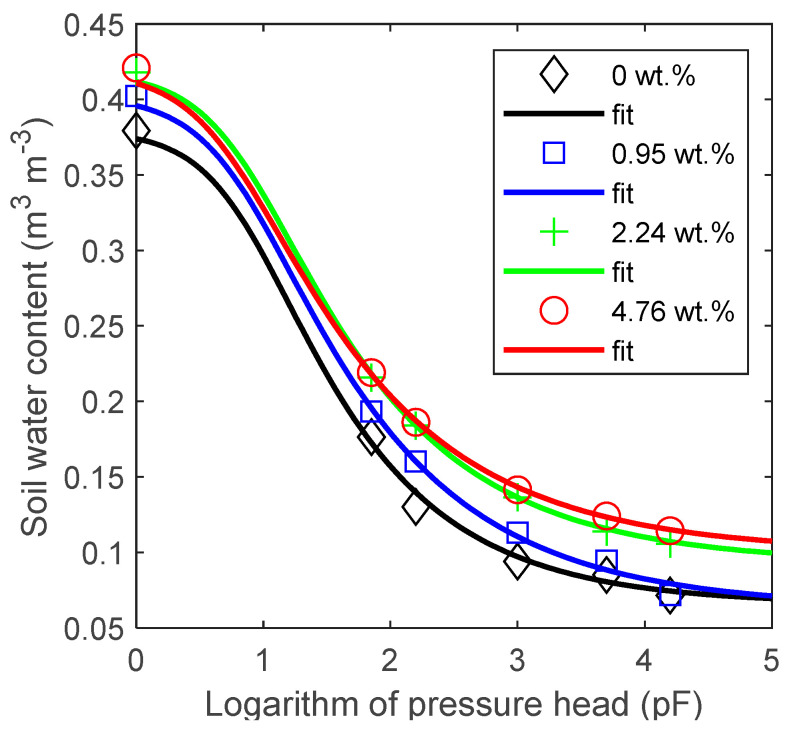
Water retention curves (symbols—experiment, solid lines—VG equation fit) of sandy soil amended with different doses (0.95, 2.24, and 4.76 wt.%) of sieved biochar (ϕ < 2000 µm). The addition of the sunflower husk biochar to sandy soil caused an increase in the value of $\theta_{S}$ (soil moisture at saturation) and in the matric suction values corresponding to the wilting point (pF = 4.2). The correlation coefficients for all fits were very close to unity.

The data points and modelled WRCs for soil samples amended with the sieved biochar (ϕ < 2000 µm), compared to the unamended reference, are shown in Figure 4.

The fitting parameters of the VG function (Equation (3)) are listed in Table 2. The results for the sieved biochar and soil mixtures show a general trend towards an increase in the soil moisture at the saturation pF = 0, field capacity pF = 2.0, and wilting point pF = 4.2, compared to the reference sand sample. Nevertheless, the VG parameters fitted to the WRC data points revealed a consistent increase only in the case of the saturation soil moisture value, $\theta_{S}$, for all doses. The introduction of the smallest amount of biochar (0.95 wt.%) into the soil caused an initial decrease in $\theta_{r}$ and n despite a further increase in the value of these parameters at higher doses. A similar pattern but in a reversed sequence was observed for α.

**Figure 5:**
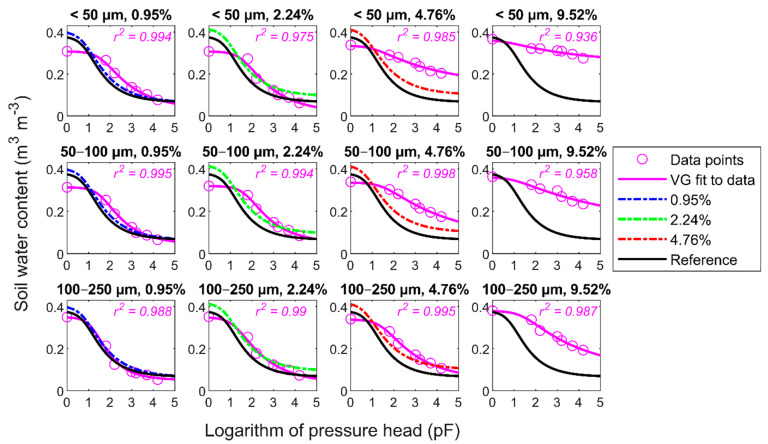
The water retention data for the fine fractions (<50 µm, 50–100 µm, and 100–250 µm) and four doses (0.95, 2.24, 4.76, and additionally 9.52 wt.%) of biochar are shown together with the van Genuchten (VG) function fit (both in magenta) as circles and solid lines, respectively. The quality of the fits is characterised by the coefficient of determination ($r^{2}$). The datasets are compared with the VG fit results for the corresponding doses of the sieved biochar (ϕ < 2000 µm—apart from the 9.52 wt.% content, for which the samples with sieved biochar were not measured) and the unamended reference data.

The WRCs data points and VG function fits for the fractionated biochar–soil mixtures are shown in Figure 5.

To emphasize the changes in WRCs induced by the application of the different fractions of biochar, these data are compared to the results for the sieved biochar (ϕ < 2000 µm) at the same contents and the reference curve from Figure 4. Additionally, the WRC results for the fractionated biochar supplemented at the very high amount of 9.52 wt.% are shown to supplement the evolution of hydro-physical properties across the biochar contents and fractions. The VG parameter fitting results are listed in Table 2. Similarly, as in the case of sieved biochar, we observed significant changes in the values of the VG parameters, starting from the lowest dose compared to the reference. However, in contrast to the sieved biochar case, the $\theta_{S}$ values dropped after the biochar application. This effect was the clearest at the lowest dose, but it weakened at the highest doses (horizontal direction of Figure 5). As regards the same doses (vertical direction of Figure 5), the $\theta_{S}$ values increased with the increasing biochar particle size range. Clearly, the WRC of samples containing the fractionated biochar in Figure 5 flattened with the increasing dose. The flattening of the curves was more evident for the smaller particles of the introduced biochar. This resulted from the increasing amount of residual water content represented by $\theta_{r}$ that is not available for plants. Interestingly, the $\theta_{r}$ values fitted to the data showed zero or nearly-zero values for the most flattened WRCs, and it was corroborated by the shift in the air entry suction (α) value to higher pressures.

### 3.3. Available Water Content

**Figure 6:**
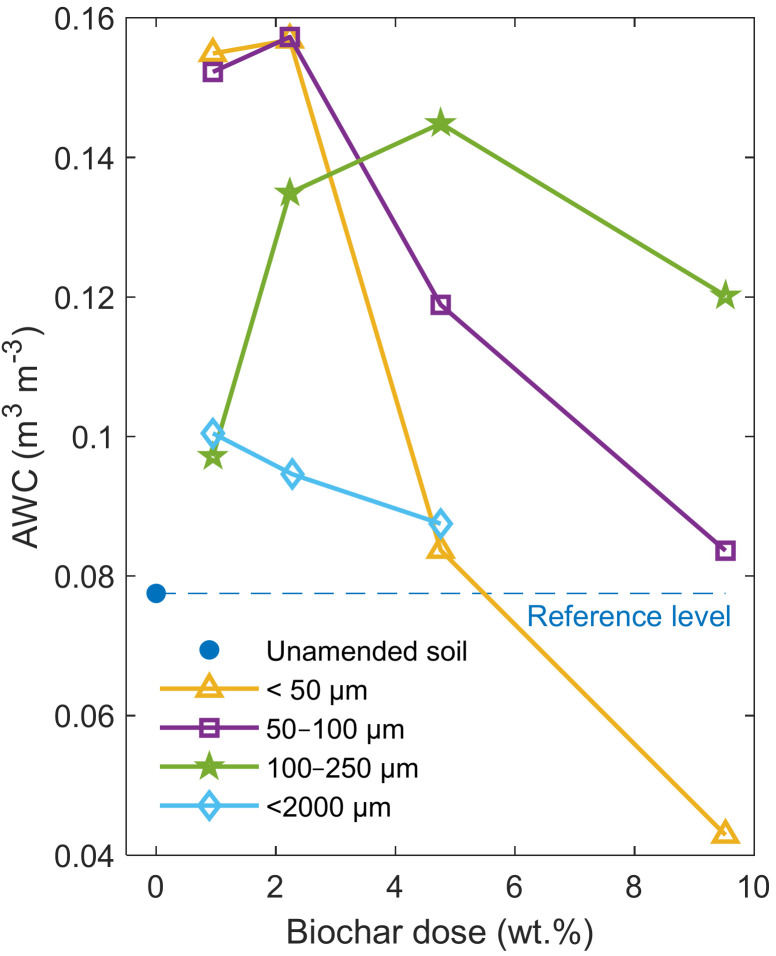
Available water content (AWC) of the investigated soil-biochar mixtures compared to the unamended sandy soil results. The sunflower husk biochar caused an increase in the AWC value at almost all doses and fractions. The most significant changes were observed for the finest size fractions below 100 µm.

As can be seen in Figure 6, after the application of the biochar, the AWC value increased for almost all biochar doses and fractions.

However, depending on the size fraction and dose, the data revealed different characteristics. Initially, all the applied biochar fractions increased the AWC in the samples, compared to the soil without biochar. After the sieved biochar amendment (ϕ < 2000 µm), AWC increased by about 0.023 m^3^ m^−3^ (30%) at the 0.95 wt.% dose to the maximum value for this fraction of 0.1 m^3^ m^−3^. The further increase in the dose induced a decrease in AWC from this value to 0.088 m^3^ m^−3^ at 4.76 wt.% (but still above the reference AWC value of 0.078 m^3^ m^−3^). The 100–250 µm fraction size caused a similar increase in the AWC value (25%), compared to the sieved biochar, only at the 0.95 wt.% biochar dose. The AWC increased to 0.145 m^3^ m^−3^ at 4.76 wt.% of biochar, which represented 87% of the reference value. However, at the highest biochar dose (9.53 wt.%), the AWC reduced its value to 0.120 m^3^ m^−3^.

In the case of the finer fractions (50–100 µm and <50 µm), the smallest dose caused a significant increase in AWC to 0.152 m^3^ m^−3^ and 0.155 m^3^ m^−3^, respectively. The increase the dose to 2.24 wt.% resulted in only a slight increase in the AWC values reaching 0.157 m^3^ m^−3^, which is a doubled value for the reference AWC of the sandy soil sample. As opposed to the 100–250 µm fraction, the 4.76 wt.% dose of both finer-size biochar caused a decrease in AWC. The highest biochar dose (9.53 wt.%) in the soil resulted in a further reduction in the AWC values for all three fine fractions, whereas the ϕ < 50 µm fraction induced a significant drop in AWC below the reference level.

## 4. Discussion

In this study, we have shown that the application of sunflower husk biochar into arable sandy soil can improve its water retention properties. This effect can be enhanced by choosing a certain biochar particle size range. The biochar in the present study revealed a fully amorphous structure (from XRD) and porous morphology (from SEM) with porosity at a moderate level (15.5%) despite the high pyrolysis temperature (650 °C). Figure 2b shows that the studied biochar contains a small number of pores holding water that is not available for plants (below 0.2 µm) [36]. Typically, biochars applied in studies on water retention improvement in soil have porosity above 70% [21,26]. The sunflower husk biochar contains a high amount of carbon equal to 85%, which is common for the relatively high pyrolysis temperature [37]. Considering this value, together with the relatively low porosity, carbon is well condensed in this pyrolysis product [38].

The bulk density of the soil-biochar samples revealed nonlinear dependencies with the clear peak values. Typically, biochar amendment causes a decrease or no significant changes in the bulk density of sandy soils [4,13,14,22,26]. In our case, the sunflower husk biochar, and especially its fine size fractions (below 100 µm) and low doses, modified the soil structure by filling the free volume between the coarse sand particles in the soil matrix.

From the hydro-physical point of view, the amendment of the sieved biochar with a grain size below 2000 µm induced the maximum increase in AWC in the biochar-soil mixtures by 0.023 m^3^ m^−3^ at the 0.95 wt.% biochar dose. Abel et al. [21] showed an approximately 0.1 m^3^ m^−3^ increase in AWC after introduction of 1 wt.% of maize biochar (0–2 mm size fraction) into sandy soil with 0.1 wt.% OM content. In turn, this increase for sandy soil with 1 wt.% OM content was approximately 0.05 m^3^ m^−3^. A wide fraction size range (0–2 mm) was also studied by Martinsen et al. [22], who derived a linear regression model with an increase in AWC of about 0.007 m^3^ m^−3^ (interpolated value) for 1 wt.% in sandy soil. Quin et al. [26] reported that 1.0 wt.% of biochar caused an increase in AWC of sand by about 0.01 m^3^ m^−3^. In turn, De Jesus Duarte et al. [13] showed an increase in AWC by 0.06 m^3^ m^−3^ at 0.461 wt.% of the biochar content in tropical forest sandy soil. However, the latter results were obtained for a tailored biochar particle size range above 250 µm and 150 µm, respectively. The source of the increase in AWC at 0.95 wt.% in our sandy soil samples is the increase in the water content at pF = 0 (saturation level) and pF = 2.0 in relation to the control and the absence of changes in the water content at the wilting point (pF = 4.2). This can be translated into the development of ultra-micropores (0.1–5 µm) and micropores (5–30 µm) in the soil [39]. It can occur through the modification of the soil structure matrix by biochar particles that can absorb the water strictly due to its porous character (biochar intrapores). Besides, the AWC value can be also increased by the modification of sand interpores with the fine biochar fraction, thereby narrowing the small micropores or mesopores (30–75 µm) to the pore size that can contribute to the AWC range (0.2–30 µm range corresponds to pF = 4.2 and 2.0, respectively from the capillary-rise equation). At the sieved biochar doses above 1 wt.%, we observed a decrease in the AWC value, whereas AWC at 4.76 wt.% of biochar was higher than the reference value only by 0.01 m^3^ m^−3^. Despite the doubling of the biochar dose from 2.24 to 4.76 wt.%, there was no significant difference in the water retention curves. A nonlinear dependence between the biochar dose and AWC was observed in sandy soil with low organic matter content shown by Abel et al. [21]. On the other hand, the same authors showed systematic increases in AWC due to biochar amendment at a moderate level of organic matter. A similar biochar-induced AWC increase in sand soil was shown by Martinsen et al. [22] and Obia et al. [4]. Here, the initial AWC increase can be explained by the dominant role of fine biochar particles that lead to a narrowing of the transmission-like pores and creating better conditions for holding the water available for plants. The further increase in the amount of the large biochar fraction leads to shrinking of the volume of soil whose pores could eventually be narrowed by the fine biochar particles, thus improving the water holding ability. With the increasing dose to above 1 wt.% of biochar, there is an evidently increased role of the narrow pores at pF = 4.2, making more water inaccessible for the plants. This effect is combined with the general increase in the water content along the entire pressure range, compared to the result of the 0.95 wt.% biochar dose sample. This suggests that the residual-like pore volumes are not created at the expense of the overall transmission-like pore and AWC region-related volumes. Our results indicate that no pores with optimal AWC diameters are formed at the biochar content above 1 wt.%, presumably due to the competition of the different biochar fractions influencing the soil matrix. Considering this competition, De Jesus Duarte et al. [13] removed the finest fraction of biochar with ϕ < 150 µm from soil samples due to its high specific surface area of small pores that hold water too strongly.

In the case of application of the fractionated biochar to the sandy soil, we observed a notable change in the hydro-physical properties of the modified soil samples, compared to the sieved biochar amendment. In particular, the samples with the finest fractions (ϕ < 50 µm, 50–100 µm) exhibited a significant change by approximately 0.08 m^3^ m^−3^ at 0.95 wt.% and 2.24 wt.%, almost doubling the reference AWC value. The retention curves shown in Figure 5 indicate a decline in the saturation water content for the biochar fraction size below 50 µm (and 50–100 µm), compared to the reference. This effect is the most evident at the lowest biochar doses but declines with the increasing biochar content. This can be explained by the decreasing pore sizes of sand filled partially with the fine fraction of biochar, which was clearly corroborated by the bulk density changes. A similar explanation of the AWC increase in sandy soil was also provided by De Jesus Duarte et al. [13]. The smaller radiuses of pores contribute to the shift of the pore size distribution in samples, which improves their retention properties and AWC at the expense of macropores filled with gravitational water. With an increasing dose, the effect of partial soil pore filling with fine biochar particles is compensated by the porous character of biochar that stores water unavailable for plants. This can be inferred from the increasing soil water content value at the wilting point with the increasing biochar content for all fractions to the 9.52 wt.% biochar content. However, the effect is less visible when the larger faction size is used due to the presumably larger specific area of smaller biochar particles. In the case of the 100–250 µm biochar fraction, the effect of partial filling of the pores is diminished by biochar particle sizes that cannot effectively fill the soil interpores and shift the pore size distribution to lower capillary radiuses for such a low dose as 0.95 wt.%. On the other hand, the biochar particles have an insufficient internal pore volume to increase the AWC as sharply as demonstrated for the finer fractions. Therefore, in our case, higher amounts of this biochar fraction should be added to the soil to observe a notable AWC increase. The maximum AWC value for this fraction is recorded at 4.76 wt.%, compared to 2.24 wt.% for the finer fractions.

From an agricultural point of view, the biochar size fraction below 100 µm was the most effective in increasing AWC. An approximately 1 wt.% dose is sufficient to almost double the reference AWC (0.078 m^3^ m^−3^) in our sandy soil. For comparison, the biochar used in ref. [22] allowed doubling the initial value at a 5 wt.% biochar dose for a particle size range below 2 mm. Głąb et al. [23] showed that fine fractions of biochar with ϕ < 500 µm were the most effective in increasing the AWC of the loamy sand, compared to 500–1000 µm and 1000–2000 µm fraction size ranges. Villagra-Mendoza and Horn [25] showed the almost twofold AWC value increase for 2.5 wt.% biochar dose. Nevertheless, as demonstrated by De Jesus Duarte et al. [13], the tuning of the specific biochar properties in soil by selecting its fraction size composition may yield a more significant effect. It depends strongly on the properties of the particular biochar fractions and target soil. This was demonstrated in Abel et al. [21], where the ϕ < 2000 µm biochar fraction acted differently on similar sandy soils with varied organic matter content.

## 5. Conclusions

The present study showed the water retention properties of sandy soil amended with low-porosity sunflower husk biochar up to 9.52 wt.% content and different size fractions. It was demonstrated that AWC can be slightly increased by the application of biochar with a grain size of ϕ < 2000 µm. The highest increase was shown for the 0.95 wt.% biochar dose, whereas the AWC decreased at the higher doses. The enhancement of the AWC in the biochar-soil mixtures was observed after the application of the fine size fraction of biochar (ϕ < 100 µm), which resulted in an almost 100% change in AWC at the 0.95 and 2.24 wt.% doses. The phenomenon can be explained by partial filling of the large ultra-micropore volumes of the sand matrix by a fine biochar fraction, resulting in an increase in bulk density. This contributed to the shift of the pore size distribution to smaller capillary radiuses to a more favourable range for the AWC increase. However, the application of doses above 3 wt.% of fine biochar caused a downturn of AWC as for ϕ < 2000 µm fraction. The results show that, despite the application of low amounts of low-porous biochar, it is possible to obtain notable AWC gains in arable sandy soil by selecting a specific fraction size of the amendment. The selection can be useful in the case of other pyrolyzed biomass products that failed to improve the soil physico-chemical properties due to the compensation of effects attributed to the certain biochar size fractions. Therefore, further studies of biochar size fractions may offer wider possibilities for the application of agro-forestry residuals.

## Figures and Tables

**Table 1:** Physico-chemical parameters of the arable soil samples.

	Parameter	Soil
	Sand (2–0.05 mm)	90
Texture (%)	Silt (0.05–0.002 mm)	9
	Clay (<0.002 mm)	1
	OM_D_* (%)	1.8
	OM_T_* (%)	2.0
	pH_H20_	7.0
	pH_KCl_	6.3

* OM_D_, OM_T_—organic matter content using H_2_O_2_ digestion and Tiurin methods, respectively.

**Table 2:** Unimodal van Genuchten model parameters according to Equation (3). The averaged bulk densities of the samples and Available Water Content (AWC) values derived from the difference between soil water contents at pF = 2.0 and 4.2.

Biochar Dose	Biochar Size Fraction	Bulk Density (mean ± SD)	$\theta_{S}$	$\theta_{r}$	α	n	AWC	Δ
wt.%	µm	g cm^−3^	m^3^ m^−3^	m^3^ m^−3^	cm^−1^		m^3^ m^−3^	m^3^ m^−3^
0		1.460 ± 0.029	0.3794	0.0747	0.067	1.692	0.078	
0.95	<50	1.666 ± 0.029	0.3082	0.0594	0.017	1.432	0.155	0.077
2.24	<50	1.665 ± 0.025	0.3075	0.0597	0.015	1.559	0.157	0.079
4.76	<50	1.580 ± 0.018	0.3381	0	0.102	1.069	0.084	0.006
9.52	<50	1.523 ± 0.008	0.3662	0.0002	0.645	1.028	0.043	−0.035
0.95	50–100	1.666 ± 0.036	0.3131	0.0501	0.021	1.447	0.152	0.075
2.24	50–100	1.627 ± 0.021	0.3191	0.0637	0.017	1.411	0.157	0.080
4.76	50–100	1.563 ± 0.023	0.3386	0.0765	0.024	1.166	0.119	0.041
9.52	50–100	1.524 ± 0.017	0.3627	0	0.069	1.061	0.084	0.006
0.95	100–250	1.511 ± 0.004	0.3498	0.0684	0.029	1.946	0.097	0.020
2.24	100–250	1.512 ± 0.001	0.3516	0.0718	0.031	1.530	0.135	0.057
4.76	100–250	1.534 ± 0.003	0.3406	0.0719	0.028	1.321	0.145	0.067
9.52	100–250	1.481 ± 0.005	0.3804	0.1038	0.042	1.172	0.120	0.043
0.95	<2000	1.540 ± 0.022	0.4024	0.0628	0.151	1.392	0.100	0.023
2.24	<2000	1.440 ± 0.008	0.418	0.0949	0.126	1.435	0.095	0.017
4.76	<2000	1.327 ± 0.033	0.4209	0.1078	0.117	1.477	0.088	0.010

SD–the standard deviation, $\theta_{S}$—saturation soil water content, $\theta_{r}$—residual soil water content, α—air entry pressure, n—dimensionless parameter.

## Data Availability

The data presented in this study are available on request from the corresponding author.
